# Inequality, public health, and COVID-19: an analysis of the Spanish case by municipalities

**DOI:** 10.1007/s10198-022-01455-9

**Published:** 2022-03-09

**Authors:** Ignacio Amate-Fortes, Almudena Guarnido-Rueda

**Affiliations:** Associate Professor of Applied Economics, Department of Economics and Business, University of Almeria, Carretera de Sacramento, s/n 04120, Almeria, Spain

**Keywords:** COVID-19, Income inequality, Unemployment, Health centers, Population density, I14, I18, D63

## Abstract

The main objective of this work is to analyze whether inequality in income distribution has an effect on COVID-19 incidence and mortality rates during the first wave of the pandemic, and how the public health system mitigates these effects. To this end, the case of 819 Spanish municipalities is used, and a linear cross-sectional model is estimated. The results obtained allow us to conclude that a higher level of income inequality generates a higher rate of infections but not deaths, highlighting the importance of the Spanish National Health Service, which does not distinguish by income level. Likewise, early detection of infection measured by the number of primary care centers per 100,000 inhabitants, access to health care for the treatment of the most severe cases, unemployment as a proxy for job insecurity, climatic conditions, and population density are also important factors that determine how COVID-19 affects the population.

## Introduction

In December 2019, the first case of coronavirus was detected in Wuhan, China. Since then, and as a result of the globalization that characterizes relations between countries, COVID-19 has affected practically all the countries of the world. It is true that some countries are suffering more severely from the consequences of this pandemic. Thus, countries such as the United States, Brazil, Russia, Spain, and India are among the countries most affected by this virus. However, observation of the data shows that COVID-19 is affecting both developed and less developed countries, so it does not seem to distinguish between rich and poor countries. However, when looking at the situation within a country, does inequality affect coronavirus incidence and mortality rates? Are the municipalities with the greatest inequality the most affected by the pandemic? These and other questions are what this paper aims to address.

Since the outbreak of COVID-19, the interest of the scientific community in the analysis of the causes and consequences of this virus has increased exponentially and, among them, numerous works have been published studying the socioeconomic determinants of this pandemic. Several authors have focused their works on the relationship between inequality and pandemic. However, most of these papers have focused on analyzing the effects that COVID-19 is having on inequality [1–3]. Therefore, the main novelty presented in this paper is to analyze inequality, not as a consequence but as a cause of the COVID-19 infection and mortality rates. To this end, the case of more than 2,000 Spanish municipalities has been analyzed. After estimating by ordinary least squares in its robust version, the conclusion reached is that inequality is a determining factor in the incidence rate, but not in the mortality rate. Likewise, we find that the availability of health centers that allow access to medical care is a fundamental variable for the prevention, detection and treatment of this virus.

The article is structured in the following way: after this introduction, the theoretical framework is exposed, analyzing the studies that have been recently published about the relationship between socioeconomic variables and the COVID-19, and making special emphasis on inequality; this is followed by an analysis of the evolution of the pandemic in Spain; later, the empirical analysis is carried out where it is explained the linear model that is going to be estimated, the variables, mainly socioeconomic, that are going to be used and the results obtained are discussed; finally, the main conclusions found are detailed.

## Theoretical framework

A macro-study published in The Lancet [4] points out that poverty and socioeconomic inequality shorten lives more than hypertension, obesity, and excessive alcohol consumption, and criticizes the fact that the WHO does not include in its agenda these health determinants that are as important or more important than others that are part of its objectives and recommendations. In fact, it was not until the 1980s that researchers' interest in studying health inequality began to grow. But does income inequality have any effect on the intensity with which pandemics, in general, and COVID-19, in particular, affect the population?

For the specific case of pandemics, there is no extensive literature addressing the socioeconomic determinants of their incidence. Even so, works such as that of [5] can be highlighted, who point out that the social determinants of health affect the results of the pandemic, and thus must be taken into account to promote public health and to mitigate its serious effects. In this regard, [6] emphasize the importance of government intervention to reduce mortality from pandemic H1N1 2009. These authors conclude that there is an inverse relationship between public spending on health and the mortality rate of the virus. [7] reach the same conclusion, pointing out that the two strongly-related determinants of coronavirus cases are population size and government health spending.

On the other hand, [8] warn that while COVID-19 began to spread more rapidly and frequently among individuals in the middle and upper classes and in high-income countries, the post-pandemic scenario will show the importance of inequality in the incidence of coronavirus and in the effects it will cause in people and countries. They, therefore, recommend that the importance of the social determinants of health in mitigating the effects of the pandemic be recognized. Furthermore, [9] note the need for socioeconomic data on the sick and dead from COVID-19 because of its significant impact on the development of public health measures. For these authors, social indicators should be considered as clinical variables, in the same way as age or sex, and, therefore, should be systematically included in medical records. [1] go a step further and point out the importance of socioeconomic factors on the direct effects of COVID-19 on health and also on the indirect effects on the economy and society. They suggest that governments should take these socioeconomic factors into account to mitigate the health and social effects of this pandemic, particularly on the most marginalized groups in society. In fact, COVID-19 has increased existing inequalities and highlighted others that were perhaps less of a concern prior to the pandemic [2]. In this regard, [10], argues that, at least during the first year of the pandemic, global inequality declined because of COVID-19, since per capita income decreased more in higher income countries. Similarly, [11] state that in the case of France, Germany, Spain, Italy, and Sweden, inequality in income distribution grew in the first months of the pandemic and, from September 2020, it began to reduce as a result of public policies that would have benefited the poorest more, as is also the case in the United Kingdom, where consumer spending fell less during the pandemic in lower income households, as their income decreased less as a result of the increase in public benefits [12]. In fact, according to [13], without compensatory public policies, wage inequality, and poverty would increase in the United States for all social groups and states. On the contrary, authors such as [14] argue, for the case of Sweden that monthly income inequality increased during the pandemic as incomes among low-wage earners were reduced while middle- and high-wage earners were hardly affected, and these differences could not be compensated through social benefits. These same conclusions are reached by [15], who state for the case of Italy that during the first wave of COVID-19, low wages were more affected, although the possibility of working at home mitigated these effects. Likewise, in the case of the Canadian labor market, COVID-19 led to greater job losses and thus income losses for workers in the bottom income quartile [16].

However, what we want to study in this paper is not the consequences of the pandemic in terms of greater or lesser inequality, but rather how income inequality may have affected the incidence and mortality rates of the virus. In fact, among the socioeconomic factors that explain the different incidence of COVID-19, inequality plays a crucial role. This inequality can be studied from several prisms. Thus, [17] conclude that the disproportionate effects of this virus on African American communities are a reflection of inequality and social exclusion that existed before the COVID-19 crisis. [18] reach the same conclusions, stating that racial, economic and health inequalities have a major effect on the population infected and killed by this virus. This, according to the authors, is due to working conditions, poverty and access to health care. These same arguments are used by [19] to explain why the most economically disadvantaged are also the most vulnerable to the COVID-19. These factors include stress, comorbidities associated with poverty, and reduced access to health care. On the other hand, [20] suggest that income inequality by county in the United States positively impacts COVID-19 incidence and mortality rates. At a more general level, [21] reach the same conclusion, i.e., in the case of OECD countries, greater income inequality is associated with higher COVID-19 mortality for the four age groups they establish. Thus, income inequality is an indicator that incorporates a series of elements of socioeconomic disadvantage that can have an impact on higher incidence and mortality rates of the virus. These include precarious housing, smoking, obesity and pollution [22].

Therefore, the economic literature has focused more on analyzing inequality as a consequence of COVID-19 rather than as one of its causes. Thus, in the absence of further empirical studies, this paper aims to further analyze the effects of inequality in income distribution on COVID-19 incidence and mortality rates. Instead of performing a study by country as detailed above, it has been decided to carry it out at the municipal level for the Spanish case, following a methodology similar to that used by [20] for the case of the United States. The variables used and their specifications have been determined by the limitations of the data collected. It was decided to analyze the Spanish case because during the first wave of COVID-19, Spain led, together with Italy, the incidence and mortality rates of the virus [23]. In addition, the study of the Spanish case is due to the availability of inequality data by municipality. The situation in Spain and Italy was subsequently reproduced in the rest of Europe, showing very similar patterns of behavior, so that the results obtained for Spain could probably be extrapolated to the case of other European countries.

## Evolution of the pandemic in Spain

**Fig. 1:**
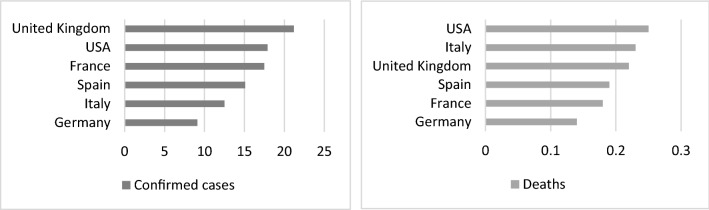
Confirmed cases and deaths per 100 population. Source: Own elaboration based on WHO data (https://covid19.who.int. Accessed on January 11, 2022)

As previously mentioned, during the first wave of COVID-19, Spain and Italy were most severely affected. These two countries became the epicenter of the pandemic in Europe. Since then, according to data from the World Health Organization,^1^ a total of 7,164,907 people have been infected in Spain and 89,934 have died. This means that confirmed cases of COVID-19 account for 15.1% of the total Spanish population, while the percentage of deaths is 0.19% of the total. As can be seen in Fig. 1, when we compare the data with other European Union countries and United States, we can see that the pandemic has hit other countries harder than the Spanish population.

**Fig. 2:**
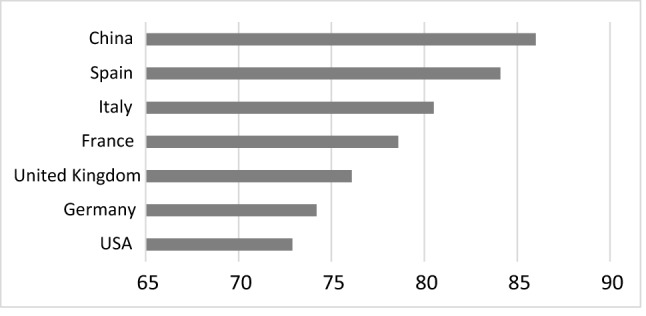
People vaccinated with at least one dose per 100 population. Source: Own elaboration based on WHO data (https://covid19.who.int. Accessed on January 12, 2022)

This evolution of the pandemic may be due to, on the one hand, the more restrictive measures imposed in Spain. The State of Alarm that was decreed on March 14, 2020 lasted for practically 14 months and involved a general confinement for 3 months, municipal confinements, curfews, etc. [24]. In fact, Italy and Spain were the only two countries that decreed the cessation of all productive activity except for essential services for 2 weeks [25]. On the other hand, the success of vaccination in Spain has softened the impact of COVID-19 on the Spanish population. As can be seen in Fig. 2, Spain is one of the countries in the world where more vaccines per inhabitant have been administered.

**Fig. 3:**
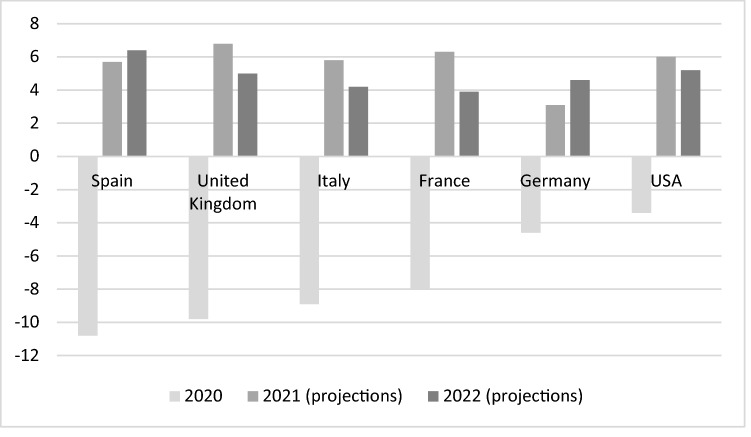
Annual percentage change in real GDP, 2020–2022. Fuente: Own elaboration based on IMF data, World Economic Outlook, October 2021 (https://www.imf.org/en/Publications/WEO/Issues/2021/10/12/world-economic-outlook-october-2021. Accessed on January 12, 2022)

From a socioeconomic point of view, Spain being one of the countries hardest hit by the pandemic at first and enacting the most restrictive measures, although it helped to reduce the epidemiological effects of COVID-19, caused a greater fall in GDP [25]. As Fig. 3 shows, Spain led the GDP decline in 2020 but also the expected recovery in 2022.

It is important to highlight that the productive structure in Spain is more dependent on sectors that have been greatly affected by the pandemic, as is the case of tourism and other in-contact intensive activities, which has made it more difficult to implement teleworking in Spain. In fact, according to Eurostat data, in 2019, only 4.8% worked at home, very low figures in relation to other countries such as the Netherlands or Finland which exceeded 14%. When the pandemic breaks out and confinement forces to work at home, in Spain telework accounted for 10.9% of the total, while in countries such as Finland, Luxembourg or Ireland it was well over 20%.

However, despite the sharp fall in GDP in Spain in 2020, this did not translate into an increase of the same magnitude in the unemployment rate. As [26] points out, the unemployment rate rose by two percentage points during 2020. This is due to a whole series of fiscal and monetary measures implemented by the Spanish government and largely financed by the European Union and the European Central Bank. To highlight some of these measures, the Records of Temporary Employment Regulation (ERTE) have made it possible to slow down the destruction of employment. Likewise, the Recovery Plan for Europe through the Next Generation EU funds, endowed with 750,000 million euros for the Member States as a whole, will mean an injection of 140,000 million euros for Spain. These funds, whose pillars are digitalization, ecological transition and reindustrialization, will be a boost and, at the same time, a challenge for the Spanish economy.

## Empirical analysis

A linear cross-sectional model has been estimated to analyze whether the income inequality observed in each Spanish municipality for which data are available has any effect on the incidence and mortality of COVID-19. The data on infection and death have been obtained from different official bodies and refer to the situation recorded on June 15, 2020, i.e., during the first wave of the pandemic and in the midst of the process of de-escalation of the lockdown that began in March. In this sense, we have worked with a database of 2319 Spanish municipalities, which represents almost 30% of all Spanish municipalities. Even so, the data on inequality by municipality has only allowed for the analysis of the case of 819 localities for the case of the study of the determinants of the COVID-19 incidence rate, and 574 localities when analyzing the mortality rate. As discussed above, the study of the Spanish case is due to the severity with which the coronavirus has affected this country.

### Data

The variables used in this work are summarized in the following Table 1.

**Table 1:** Variable definitions and summary statistics

Variable	Description	Obs	Mean	Std. Dev	Min	Max
Incidence rate	Cumulative incidence rate of COVID-19. It is defined as cases detected per 100,000 inhabitants. The data refer to the situation of contagion as of 15 June 2020. Sources: Ministry of Health, Regional Departments of Health and other official bodies. https://www.mscbs.gob.es/profesionales/saludPublica/ccayes/alertasActual/nCov/situacionActual.htm. Accessed on June 15, 2020	2319	500.8	867.7	8.1	25,490.2
Mortality rate	Cumulative number of coronavirus deaths per 100,000 inhabitants. The data refer to the situation of contagion as of 15 June 2020. Sources: Ministry of Health, Departments of Health and other official bodies. https://www.mscbs.gob.es/profesionales/saludPublica/ccayes/alertasActual/nCov/situacionActual.htm. Accessed on June 16, 2020	1180	61.3	131.3	0	2658.5
Incidence rate in the Autonomous Community	Incidence rate in the Autonomous Community where the municipality is located. The aim of this variable is to analyze to what extent the level of infection of a population depends on the level of infection of the region where it is located. Sources: Ministry of Health and Regional Departments of Health. https://www.mscbs.gob.es/profesionales/saludPublica/ccayes/alertasActual/nCov/situacionActual.htm. Accessed on June 21, 2020	2318	13.9	14.0	0.2	34.6
Average relative gross income	It is defined as the average gross income of the municipality in relation to the average gross income of the province. It is a measure of inequality that aims to study whether poorer populations within a province are more vulnerable to the effects of the pandemic. Source: Prepared by the authors based on data from the Tax Office, Ministry of Finance (2017). https://www.agenciatributaria.es/AEAT/Contenidos_Comunes/La_Agencia_Tributaria/Estadisticas/Publicaciones/sites/irpfmunicipios/2016/jrubik1ef468a251d390b847ce88908aaafc743028fb8d.html. Accessed on August, 10, 2020	1872	89.5	17.4	48.8	213.0
Inequality	Inequality index by municipality. The following five inequality indexes have been used: Gini Index. Measure of inequality in the distribution of the municipality's taxable income, calculated from the microdata of the Annual Sample of Personal Income Tax. The value of the index varies between 0 and 1. Source: [29]	892	0.47	0.06	0.24	0.78
	Atkinson Index. Calculated for an inequality aversion parameter equal to 0.5, this measure of inequality is obtained from the distribution of the municipality's taxable income, calculated from the microdata of the Annual Sample of Personal Income Tax Returnees. The value of the index varies between 0 and 1. Source1: [29]	892	0.22	0.05	0.09	0.58
	80/20 Index. A measure of inequality that relates the percentage of the municipality's aggregate taxable income obtained by the top 20% of income tax filers to the bottom 20%. Source: Own elaboration based on [29]	892	25.6	16.0	5.03	175.7
	Top 1%. A measure of income concentration that includes the percentage of the municipality's aggregate taxable income obtained by the 1% of its inhabitants filing income tax returns with the highest taxable income in the year of the statistics. Source: [29]	892	8.74	4.40	2.48	39.52
	Top 0.1%. A measure of income concentration that includes the percentage of the municipality's aggregate taxable income obtained by the 0.1% of its inhabitants filing income tax returns with the highest taxable income in the year of the statistics. Source: [29]	892	2.15	2.01	0.35	16.5
Primary care centers	Number of primary care centers of the National Health System in the municipality per 100,000 inhabitants This variable is used to determine the importance that these centers have in the early detection of the virus and, therefore, in the containment of the COVID-19 contagion. Source: Own elaboration based on data from the Ministry of Health (2020). https://www.mscbs.gob.es/ciudadanos/centrosCA.do. Accessed on June, 29, 2020	2320	78.5	119.6	0	1612.9
Hospitals	Number of public and private hospitals per municipality and per 100,000 inhabitants. The objective of the use of this variable is to determine the importance of hospitals, not so in the detection of the virus, but in the fight against the mortality of the COVID-19. Source: Own elaboration from data of the Ministry of Health, National Hospitals Catalogue (2019). https://www.mscbs.gob.es/ciudadanos/prestaciones/centrosServiciosSNS/hospitales/home.htm. Accessed on June, 30, 2020	2320	0.87	4.23	0	80.6
Unemployment	Number of unemployed registered in February 2020 (just before the confinement that took place in March) by municipality in relation to the total population. The goal is to verify if higher unemployment has any effect on the incidence and mortality rate of the virus. February data are used since March data is influenced by the economic closure that the confinement entailed. Source: Own elaboration from the data of the State Employment Public Service (Ministry of Labor and Social Economy) and the National Institute of Statistics. https://www.sepe.es/HomeSepe/que-es-el-sepe/estadisticas/datos-estadisticos/municipios/2020/febrero.html. Accessed on July 1, 2020	2295	5.89	2.57	0.01	27.4
Population density	Variable that measures the population per km2 by municipality. The use of this variable allows to check if a lower density allows to better contain the incidence of the virus as it is easier to maintain social distance. Source: Data from the register of local entities. Ministry of Finance and Public Administration (2020). https://ssweb.seap.minhap.es/REL/frontend/inicio/municipios/all/all. Accessed on June 20, 2020	2311	503.5	1476.8	1.55	21,522.7
Temperature	Variable that collects the average temperature by municipality. The aim of using this variable is to determine if the warmer areas have a lower incidence of the virus. Source: State Meteorological Agency (1981–2010) y Climate-data.org (1982–2012). http://www.aemet.es/es/serviciosclimaticos/datosclimatologicos/ and https://es.climate-data.org. Accessed on August 15, 2020	839	16.2	1.83	9.1	20.9
Public Health Expenditure	Mide el gasto público en salud por habitante en cada Comunidad Autónoma. Con ello, se pretende analizar si aquellas regiones que más gastan en salud les ha permitido luchar mejor contra el virus. Source: Ministry of Health. Public Health Expenditure Statistics (2015). https://www.mscbs.gob.es/estadEstudios/estadisticas/docs/EGSP2008/egspPrincipalesResultados.pdf. Accessed on July 7, 2020	2318	1408.9	157.4	1212	1753

Source: Own elaboration

### The model

The linear model used has been estimated through Ordinary Least Squares in its robust version of variances and covariances, since when performing the Breusch–Pagan test, the *p* value obtained has shown the presence of heteroscedasticity in the model. The model has been estimated without a constant term. Although the decision to use a constant term or not is a problem that generates much discussion [27], nevertheless, there are circumstances in which it is appropriate or even necessary not to use the constant term. As [28] points out, in the case where the dependent variable is zero if the vector of independent variables is also zero, the constant term can be omitted. This is the case of the estimated model where variables such as the incidence rate of the autonomous community where the municipality in question is located or population density are used. If these variables had a value equal to zero, the COVID-19 incidence and mortality rates would be zero.

The model used is the following:

1 $$\begin{gathered} {\text{COVID }}19 = \beta_{1} {\text{INCIDENCE}} + \beta_{2} {\text{INCOME}} + \beta_{3} {\text{INEQUALITY}} \hfill \\ \quad \quad \quad \quad \quad + \beta_{4} {\text{HOSPITALS}} + \beta_{5} {\text{UNEMPLOYMENT}} + \beta_{6} {\text{DENSITY}} \hfill \\ \quad \quad \quad \quad \quad + \beta_{7} {\text{TEMPERATURE}} + \beta_{8} {\text{EXPENDITURE}} + \mu_{I} \hfill \\ \end{gathered}$$

where* COVID19* is the dependent variable. In this sense, two variables have been used, on the one hand, the COVID-19 incidence rate and, on the other, the mortality rate. In both cases it has been measured per 100,000 inhabitants. The aim is to analyze at first how the independent variables used affect the rate of infection of this virus and, subsequently, check how these effects vary on the mortality rate. It is important to mention that the data used refer to the first wave of the pandemic in Spain and are the accumulated data by municipalities until June 15, 2020.

*INCIDENCE* is the incidence rate in the Autonomous Community where the municipality is located. This variable is used to verify the importance of community contagion within the regions. In this case, the data are also those accumulated as of June 15, 2020.

*INCOME* refers to the average gross income of the municipality in relation to the average gross income of the province. It is, therefore, a first variable that measures inequality, in this case, among municipalities. This variable is used to analyze the extent to which a higher relative income of the municipality may affect the incidence and mortality rate of the COVID-19. The data we have been able to use are those referring to 2016. In any case, to our understanding, the relative incomes between municipalities and provinces hardly undergo significant changes, and even less in a time span of 4 years.

*INEQUALITY* is the explanatory variable on which we have focused the objective of this work, that is, we intend to analyze how inequality within each municipality affects both the rate of infection and the mortality rate of the coronavirus. To this end, five measures of inequality have been used: the Gini index, the Atkinson index, the 80/20 ratio, and the concentration of income in the richest 1% and 0.1%. The data have been obtained from the work of [29] and, therefore, refer to 2014. However, as we have commented above, income inequality undergoes very small changes in short periods of time.

*HOSPITALS* is a proxy variable of the human and physical health resources available to the municipality. In this sense, two variables have been used. On the one hand, the number of primary care centers per 100,000 inhabitants is used, since these are the ones in charge of detecting, in the first instance, the presence of coronavirus. Therefore, we want to check to what extent the number of primary care centers affects the rate of incidence of COVID-19. On the other hand, the number of hospitals per 100,000 inhabitants is used to analyze its effect on the mortality rate, since hospitals are in charge of treating the most severe cases. In this case, the database used refers to the situation in 2020 for the case of primary care centers and 2019 for the number of hospitals.

*UNEMPLOYMENT* measures the number of registered unemployed in February 2020, that is, before the lockdown that took place in Spain in March, in relation to the total population of the municipality. The unemployment rate is not used due to lack of data. The aim of using this variable is to analyze whether higher unemployment has any effect on the rate of contagion and mortality of the virus.

*DENSITY* is the population density of the municipality in 2020, i.e., number of inhabitants per km2. The use of this variable has a double objective. On the one hand, it is intended to check whether the larger municipalities, which tend to have a higher population density, have been more exposed to contagion. And, on the other hand, to verify the importance of social distance in the fight against this pandemic.

*TEMPERATURE* measures the average temperature of the municipality. This variable is used to study whether it is easier to be infected in cold areas than in warm ones. For this variable, the average data recorded in each municipality between 1982 and 2012 was used.

*EXPENDITURE* includes public spending on health per inhabitant. The aim of using this variable is to analyze whether a higher health expenditure by the Autonomous Community in which the municipality is located has had any effect on the incidence rate and mortality rate of the COVID-19. The data that could be used for this variable refer to the situation in 2015. We understand that the changes that may have occurred in the last 5 years have not been large enough to alter the results.

## Results

As mentioned above, the model has been estimated by OLS in its robust version to solve the problem of heteroscedasticity detected. Fifteen estimates have been made, which are the result of the use of five different measures of inequality, as well as the employment, on the one hand, of the primary care centers and, on the other, of the hospitals. The results are presented in the following Tables 2, 3 and 4:

**Table 2:** Results of the estimations (incidence rate)

	Gini	Atkinson	80/20	Top 1%	Top 0.1%
Incidence rate in the Autonomous Community	12.95*** (8.47)	12.88*** (8.38)	12.84*** (8.32)	12.89*** (8.32)	12.88*** (8.24)
Average relative gross income	1.30** (2.20)	1.61*** (2.76)	1.89*** (3.31)	1.70*** (2.93)	1.81*** (3.02)
Inequality	5.39*** (3.17)	4.58** (2.50)	0.29 (0.71)	4.41* (1.83)	3.53 (0.69)
Primary care centers	– 2.95*** ( – 3.01)	– 2.75*** ( – 2.87)	– 2.58*** ( – 2.72)	– 2.53*** ( – 2.67)	– 2.50*** ( – 2.60)
Unemployment	12.91*** (2.93)	13.69*** (3.08)	13.86*** (3.06)	15.81*** (3.57)	14.79*** (3.35)
Population density	0.02*** (3.52)	0.02***(3.38)	0.02*** (3.25)	0.02*** (3.30)	0.02*** (3.25)
Temperature	– 7.99*** ( – 6.72)	– 7.65*** (−6.68)	– 7.40*** ( – 6.49)	– 7.47*** ( – 6.63)	– 7.40***( – 6.52)
Public health expenditure	0.81*** (5.93)	0.86*** (6.12)	0.87*** (6.26)	0.86*** (6.05)	0.87*** (6.24)
Number of observations	819	819	819	819	819
R^2^	0.66	0.65	0.65	0.65	0.65

*Significant at 10%, **Significant at 5%, ***Significant at 1%

**Table 3:** Results of the estimations (mortality rate) (1)

	Gini	Atkinson	80/20	Top 1%	Top 0.1%
Incidence rate in the Autonomous Community	3.67*** (13.45)	3.69 (13.53)	3.66*** (13.57)	3.66*** (13.59)	3.66*** (13.58)
Average relative gross income	– 0.36** ( – 3.25)	– 0.37*** ( – 2.76)	– 0.34*** ( – 3.17)	– 0.36*** ( – 3.44)	– 0.36*** ( – 3.55)
Inequality	2.51 (0.54)	8.15 (1.48)	0.11 (0.81)	0.72 (1.37)	0.76 (0.60)
Primary care centers	– 0.19 ( – 1.14)	– 0.21 ( – 1.25)	– 0.18 ( – 1.06)	– 0.17 ( – 1.00)	– 0.16 ( – 0.93)
Unemployment	1.01 (1.02)	1.07 (1.08)	0.97 (0.96)	1.32 (1.34)	1.13 (1.13)
Population density	0.002** (2.44)	0.002*** (2.58)	0.002** (2.48)	0.002** (2.48)	0.002** (2.45)
Temperature	– 6.22*** ( – 3.37)	– 6.51*** ( – 3.68)	– 6.08*** ( – 3.51)	– 6.06*** ( – 3.56)	– 5.91*** ( – 3.45)
Public health expenditure	0.10*** (4.41)	0.10*** (4.49)	0.10*** (4.76)	0.10*** (4.54)	0.10*** (4.75)
Number of observations	574	574	574	574	574
*R*^*2*^	0.74	0.75	0.74	0.75	0.74

*Significant at 10%, **Significant at 5%, ***Significant at 1%

**Table 4:** Results of the estimations (mortality rate)

	Gini	Atkinson	80/20	Top 1%	Top 0.1%
Incidence rate in the Autonomous Community	3.80*** (13.68)	3.82*** (13.79)	3.79*** (13.83)	3.80*** (13.83)	3.79*** (13.82)
Average relative gross income	– 0.27*( – 2.55)	– 0.28***( – 2.70)	– 0.26**( – 2.48)	– 0.28***( – 2.75)	– 0.28***( – 2.73)
Inequality	1.68 (0.37)	6.90 (1.28)	0.09 (0.64)	0.63 (1.21)	0.94 (0.75)
Hospitals	– 2.52*** ( – 2.58)	– 2.47** ( – 2.52)	– 2.51*** ( – 2.58)	– 2.48** ( – 2.55)	– 2.53 ( – 2.61)
Unemployment	1.36 (1.37)	1.41 (1.42)	1.32 (1.31)	1.61 (1.63)	1.49 (1.48)
Population density	0.002*** (2.67)	0.002*** (2.86)	0.002*** (2.78)	0.002*** (2.79)	0.002*** (2.76)
Temperature	– 6.64*** ( – 3.65)	– 6.94*** ( – 3.99)	– 6.56*** ( – 3.87)	– 6.56 ( – 3.93)	– 6.44*** ( – 3.84)
Public health expenditure	0.10*** (4.40)	0.10*** (4.44)	0.10*** (4.7)	0.10*** (4.50)	0.10*** (4.70)
Number of observations	574	574	574	574	574
*R*^2^	0.75	0.75	0.75	0.75	0.75

*Significant at 10%, **Significant at 5%, ***Significant at 1%

On the other hand, and to check the robustness of the model, and as other authors have done [30], it was estimated by excluding from the sample the 5% of the highest and lowest municipalities in terms of incidence rate and mortality rate for the dependent variables, and population density for the regressors. In this case, only the Gini index was used as a measure of inequality. The results of the robustness check of the model are shown in the following Table 5:

**Table 5:** Robustness check of the model

	Incidence rate	Mortality rate
Incidence rate in the Autonomous Community	11.38*** (11.03)	2.96*** (12.65)
Average relative gross income	1.64** (2.95)	– 0.14 ( – 1.54)
Inequality	6.13*** (4.30)	11.20 (0.34)
Primary care centers	– 2.12* ( – 1.99)	– 0.10 ( – 0.55)
Unemployment	10.52** (2.74)	– 0.32 ( – 0.41)
Population density	0.05*** (5.29)	0.005*** (3.34)
Temperature	– 6.59*** ( – 10.82)	– 4.67*** ( – 2.66)
Public health expenditure	0.57*** (7.78)	0.10*** (3.60)
Number of observations	741	514
*R*2	0.76	0.77

*Significant at 10%, **Significant at 5%, ***Significant at 1%

The first conclusion that can be drawn from the 15 estimates is that the model is robust since there are hardly any significant changes in either the estimated regressors or their significance. In addition, the robustness check carried out shows that the model is robust. Likewise, the quality of the adjustment is good since the R^2^ ranges from 0.65 (Tables 2) to 0.75 (Tables 3 and 4).

As for the values obtained, in most cases they are those expected a priori. Thus the parameter estimated for the rate of incidence of COVID-19 in the Autonomous Community where the municipality is located is positive and highly significant in all estimates, that is, the higher the level of infection within the region, the greater the rate of incidence and mortality of the virus in the municipality. There is a logical difference, the effect is stronger on the incidence rate than on the mortality rate, that is, the latter is influenced by other factors than the mere level of contagion that may exist in the region.

Focusing now on the fundamental objective of this work, which is to analyze how inequality affects the incidence and mortality rates of the coronavirus, the first measure used is the average gross income of the municipality in relation to that of the province in which it is located. The positive and significant sign observed in Table 2 shows a positive relationship between this variable and the virus incidence rate, that is, those populations that are richer in relative terms and probably more populated are more exposed to the COVID-19 infection. However, the parameter of this variable changes sign when we use as dependent variable the mortality rate (Tables 3 and 4). However, the parameter of this variable changes when we use the mortality rate as a dependent variable (Tables 3 and 4). In our opinion, this is due to the reduced availability of hospital centers in these types of municipalities, fundamentally if they are small rural municipalities where, in addition, there is a greater proportion of elderly people.

However, whether a municipality is rich or poor does not mean that there is more or less inequality. Thus, deepening the analysis on the effects that inequality has on COVID-19 incidence and mortality rates, five measures of inequality have been used for each municipality. The aim is to see if greater inequality within the municipality has an effect on the level of contagion and mortality of this virus. In the case of the incidence rate (Table 2), the five estimates present a positive estimated parameter, although only significant in three of the cases, that is, when using the Gini index, the Atkinson index and the concentration of income in the top 1% of the population. Thus, it can be concluded that greater income inequality, this time within municipalities, leads to a higher level of infection, since this greater inequality shows that a significant part of the population does not have the necessary means to follow COVID-19 prevention measures, result also reached by [20]. In this sense, precarious labor conditions, the stress this entails, and the comorbidities associated with poverty that may characterize these more unequal municipalities could explain these results as do other authors [18, 19, 22]. However, although the sign remains positive, the significance is zero when we estimate the effect that inequality has on the mortality rate of the virus (Tables 3 and 4). This implies that inequality is not a determining factor in the mortality rate of COVID-19, highlighting the importance of having a good public health system. These results support the conclusions of [31] who argue that, although the frequency of hospitalization or the use of emergency services does not differ greatly by income level, curative and preventive services do. This different effect of income inequality on COVID-19 incidence and mortality rates is the main conclusion of the study. This shows that although the virus does distinguish between rich and poor, the Spanish National Health System, which is responsible for treating the most serious cases through hospital treatment, does not differentiate by income level and makes it an effective mechanism for reducing inequalities in society.

In this sense, a variable reflecting the number of primary care centers per 100,000 inhabitants has been included in the model. As can be seen in Table 2 (incidence rate as a dependent variable), the estimated sign for this variable is always negative and highly significant. This result implies that the greater the number of primary care centers, the lower the incidence rate of coronavirus. This result highlights the importance of these centers since they are the first to be attended by people who feel the symptoms and, therefore, are essential for the early detection of the virus and, in this way, avoid further contagion of it. However, when we check the effect that the number of primary care centers has on the mortality rate (Table 3), the parameter obtained is not significant in any of the five estimates made. This result is logical since death as a result of COVID-19 is preceded by a worsening of the disease that cannot be treated in these centers. That is why we decided to re-estimate the model by replacing this variable with another one that takes into account the number of hospitals per 100,000 inhabitants. Table 4 shows that the sign of the parameter estimated for this new variable is negative and significant in four of the five estimates, thus demonstrating that the greater the number of hospitals, the more effective the fight against the mortality caused by the coronavirus. Therefore, access to health services is a fundamental element, as also argued by [18, 19]. However, contrary to [6], this does not imply that more should be spent on health. In fact, the estimated regressor for the variable that collects the public expenditure on health per inhabitant shows a positive and significant sign, so that the municipalities of the Autonomous Communities that spend more on health are not the ones that suffer a lower incidence and mortality rate. This shows that it is not a question of spending more but rather of spending better, as shown by [32].

As for the "Unemployment" variable, the results obtained emphasize the conclusions reached from the analysis of inequality as a determining factor in the incidence and mortality rates of COVID-19. Thus, the sign of the estimated regressor for this variable is always positive, although it is only significant when we use the virus incidence rate as a dependent variable (Table 2). This implies that the higher the unemployment in a municipality, the more precarious the employment and the more difficult it is to implement teleworking, and thus the population is more exposed to contagion than in other municipalities where unemployment is lower. Factors already pointed out such as stress [19] and social exclusion [17] make unemployment a very important determinant of the incidence of COVID-19. In fact, unemployment has very negative effects on health [33].

Therefore, prevention measures are the best instrument to fight this virus. In this sense, the population density shows the expected effect a priori, that is, the estimated sign for this variable is always positive and significant, so that in those municipalities where the population density is lower, the rate of infection and death rate is also lower because it is easier to maintain social distance. Likewise, taking into account that the largest municipalities tend to have the highest population density, this result shows that it is the largest cities that are most affected by this virus. These results are consistent with those obtained by [7, 34].

Finally, the scientific community has discussed about the climatic conditions and its effects on the level of infections and deaths produced by the COVID-19. It is observed that this virus is affecting practically all countries, both those with cold climates and those with warmer ones. The question here is whether temperature is a determining factor in the spread of the virus within a country. The estimated sign for this variable is always negative and highly significant, so we can say that those municipalities with higher average temperatures suffer a lower incidence and mortality of the virus. This result confirms the theses of other authors such as [35, 36], contradicting the results of other studies such as [37, 38].

## Conclusions

Does income inequality matter to COVID-19? This and other questions have been addressed by this paper. The empirical analysis undertaken shows that inequality is a key element to explain the differences in the incidence rates of coronavirus among Spanish municipalities. The polarization of the income distribution, with a high percentage of the population located in the lower income levels, affected by higher unemployment or greater job insecurity, which makes it more difficult to implement teleworking and, therefore, to achieve social distancing, makes the fight against inequality a key policy for the prevention of this type of pandemic.

In fact, prevention is the first element in the fight against the coronavirus. The population density of the studied municipalities has a direct effect on the incidence and mortality rates of the COVID-19, so ensuring social distancing, as it was achieved during the 3 months of lockdown in Spain, should be the first measure to be adopted.

This work shows that primary care centers, which are the first health care facilities that people go to when they feel the symptoms of the coronavirus, are a key element in the fight against the pandemic. Having a sufficient number of primary care centers that allow adequate access to health services and avoid health collapse should be a fundamental objective for the Autonomous Communities that have the competences in health policy.

Access to health treatment for the most severe cases should be the last element to be addressed to reduce, in this case, the mortality rate of the COVID-19. Inequality in income distribution has no significant effect on the number of deaths from the coronavirus in relation to the population. This implies that, once infected, the Spanish National Health System does not distinguish between rich and poor. Therefore, this work highlights the importance of having a good network of hospitals, since these are the ones in charge of treating the most severe patients. Once again, adequate access to health services is fundamental in the fight against this virus. In this sense, the results reflect that the municipalities of the Autonomous Communities that spend more on health care are not the least affected by the COVID-19, which leads us to conclude that it is not a question of spending more but of spending better, more efficiently, that is, investing the health budget in facilitating access to the medical care that the population requires at each moment.

Nevertheless, this study has several limitations. First, the data did not allow us to divide the sample by age group or gender, which would have increased the usefulness of the results. Likewise, it would have been interesting to have data by municipality that would have allowed us to construct ratios of inequality 90/10 or 75/25. This would have served to check the robustness of the model. Finally, although there is a growing literature that tries to relate COVID-19 to working from home [39–41], however, it has not been possible to work with a database for this variable by municipality or province. It would have been interesting to have been able to include this variable in the model as this would have strengthened the conclusions on the effects of working conditions on COVID-19 incidence and mortality rates.

## Footnotes

1 https://covid19.who.int Accessed on January 11, 2022.
